# Low level exposure to cadmium increases the risk of chronic kidney disease: analysis of the NHANES 1999-2006

**DOI:** 10.1186/1471-2458-10-304

**Published:** 2010-06-03

**Authors:** Pietro Manuel Ferraro, Stefano Costanzi, Alessandro Naticchia, Antonio Sturniolo, Giovanni Gambaro

**Affiliations:** 1 Division of Nephrology and Dialysis, Columbus-Gemelli University Hospital, Renal Program, Catholic University, Rome, Italy

## Abstract

**Background:**

Environmental factors have been associated with the outbreak of chronic kidney disease (CKD). We evaluated the association of Cadmium (Cd) exposure with the risk of CKD in U.S. adults who participated in the 1999-2006 National Health and Nutrition Examination Surveys (NHANES).

**Methods:**

5426 subjects ≥ 20 years were stratified for values of urinary and blood Cd and a multivariate logistic regression was performed to test the association between blood and urinary Cd, CKD and albuminuria (ALB) after adjustment for age, gender, race/ethnicity, body mass index and smoking habits.

**Results:**

Subjects with urinary Cd > 1 mcg/g and subjects with blood Cd > 1 mcg/L showed a higher association with ALB (OR 1.63, 95% CI 1.23, 2.16; *P *= 0.001). Subjects with blood Cd > 1 mcg/L showed a higher association with both CKD (OR 1.48, 95% CI 1.01, 2.17; *P *= 0.046) and ALB (OR 1.41, 95% CI 1.10, 1.82; *P *= 0.007). An interaction effect on ALB was found for high levels of urinary and blood Cd (*P *= 0.014).

**Conclusions:**

Moderately high levels of urinary and blood Cd are associated with a higher proportion of CKD and ALB in the United States population.

## Background

Chronic exposure to Cadmium (Cd), an industrial and environmental pollutant, can cause both renal proximal tubular damage and decline in glomerular filtration rate (GFR) in humans; this has been confirmed in experimental models [1,2]. After pulmonary and/or gastrointestinal absorption, Cd binds to serum albumin and accumulates in the liver, where it is complexed to a metal-binding protein with a high affinity for Cd, metallothionein-1 [3]. The Cd-metallothionein-1 complex reaches the kidney where it is filtered and accumulates in the proximal tubule, whose cells possess transporters for free and bound forms of Cd [4] and interferes with the tubular function.

Cadmium nephropathy is characterized by low molecular weight (LMW) proteinuria due to diminished intrarenal uptake and catabolism of filtered proteins. In Cadmium nephropathy, proximal tubular dysfunction persists until renal failure supervenes.

Although Cadmium nephropathy has been observed in workers exposed to high levels of Cd, recent data suggest that relatively low levels of exposure in people living in polluted industrial areas increase the risk of tubular dysfunction [1]. Furthermore, analysis of the National Health and Nutrition Examination Survey (NHANES) databases have disclosed even in the general population the association of Cd exposure with some clinical conditions [5-7]. In particular, the very recent paper by Navas-Acien et al [8] has disclosed that marginally high levels of blood Cd are significantly associated with kidney dysfunction. However, blood Cd is considered to be a marker of the acute, short term exposition, rather than the chronic one which is much more relevant to Cadmium nephropathy [9]. Since the urinary excretion of Cd is assumed to mirror chronic exposition to Cd, and Navas-Acien did not investigate this topic, we have specifically addressed whether an association exists between urinary Cd and renal dysfunction.

We analyzed the association between blood and urinary Cd levels and kidney abnormalities (chronic kidney disease [CKD] and albuminuria [ALB]) among U.S. adults who participated in the 1999-2006 NHANES surveys to support the idea that Cd exposition of the general population contributes to the epidemics of CKD in this population.

## Methods

The NHANES is a cross-sectional nationally representative survey of the noninstitutionalized U.S. civilian population [10], and is regularly conducted in the U.S. by the National Center for Health statistics. It consists of standardized questionnaires administered in the home by trained interviewers followed by a detailed physical examination at a Mobile Examination Center. Data from the 1999-2000, 2001-2002, 2003-2004 and 2005-2006 NHANES surveys were available from the website http://www.cdc.gov/nchs/nhanes.htm[10]. We limited our analysis to subjects ≥ 20 years of age. Data relevant to the analysis included age, gender, race/ethnicity, body mass index (BMI), self-reported information about smoking habits.

A blood specimen was obtained from participants for blood Cd and creatinine. An untimed urine sample was obtained from a random subsample of participants for urinary Cd, albumin and creatinine.

Exclusions for the current study included subjects without the measurements necessary for our analysis, and pregnant or menstruating women at the time of examination.

Cadmium concentrations were determined at the Environmental Health Sciences Laboratory of the CDC National Center for Environmental Health (NCEH) after confirmation of no background contamination and using extensive quality control procedures. For blood Cd measurements, a PerkinElmer Model SIMAA 6000 simultaneous multi-element atomic absorption spectrometer with Zeeman background correction was used. Urinary Cd concentrations were determined using inductively coupled plasma mass spectrometry.

The limit of detection for urinary Cd was 0.04 mcg/L; of the study participants, 1.2% had urinary Cd levels below this limit.

The limit of detection for blood Cd was 0.30 mcg/L in NHANES 1999-2002 and 0.20 mcg/L in NHANES 2003-2006: 17.0% and 5.3% of the study participants had blood Cd levels below the limits of detection in NHANES 1999-2002 and NHANES 2003-2006, respectively.

In our analysis urinary Cd levels have been expressed as a urinary Cd-to-creatinine ratio (mcg/g creatinine).

Serum and urinary creatinine concentrations were determined using the Jaffe kinetic alkaline picrate method. Estimated GFR (eGFR) was calculated from standardized serum creatinine using the CKD-EPI study equation [11].

Since proper use of GFR estimating equations requires a known calibration of the serum creatinine assays [12], unstandardized serum creatinine from the 1999-2000 survey was corrected applying the following formula, available from the NHANES website [10]:

Uncalibrated serum creatinine from the 2005-2006 survey has been corrected using the formula [10]:

No creatinine calibration was needed for the 2001-02 and 2003-04 surveys values.

Urinary albumin concentration has been determined using a solid-phase fluorescent immunoassay.

CKD has been defined as an eGFR < 60 mL/min/1.73 m^2^; ALB as a urinary albumin-to-creatinine ratio (UACR) ≥ 20 mg/g for males and ≥ 30 mg/g for females; smoke by self-reported data: participants who reported having smoked ≥ 100 cigarettes during their lifetime were classified as smokers.

Significant differences in the relative frequencies of CKD and ALB have been calculated by means of a multivariate logistic regression model including age, gender, race/ethnicity, BMI and smoking habits.

Continuous variables were tested for normality of distribution by means of numerical methods (skewness and kurtosis) and all resulted non normally distributed, so their values were reported as median (interquartile range). Categorical variables were reported as total number (percent). Linear relations between continuous variables have been evaluated by Spearman correlation. An eGFR < 10 mL/min or > 130 mL/min have not been considered for linear analysis. Mann-Whitney and Kruskal-Wallis tests have been performed for comparison of continuous variables between two or more groups, respectively. When using the Kruskal-Wallis test, differences between groups have been carried out by Mann-Whitney tests for pairs of groups if the overall F was significant. For comparisons between categorical variables the Chi-square test has been used. The accepted level for a two-tailed significant difference was *P *< 0.05. All statistical analyses were performed by using the survey package in the R statistical language to account for the complex sampling design and weights in NHANES surveys. Figures have been obtained with GraphPad Prism version 5.0 (GraphPad Software, San Diego, CA, USA).

The NHANES survey is subject to the CDC/NCHS Ethics Review Board (ERB) to ensure that appropriate human subjects protections are provided, in compliance with 45 CFR part 46.

## Results

The overall analysis included 5426 subjects, whose characteristics are shown in Table 1.

**Table 1:** Characteristics of the study population

Age (years)	47 (32)
Males	2644 (48.7%)

Race/ethnicity	
- Mexican American	1226 (22.6%)
- Non Hispanic White	2740 (50.5%)
- Non Hispanic Black	1072 (19.8%)
- Other	388 (7.2%)

BMI (Kg/m^2^)	27.5 (7.4)

Smokers	2649 (48.8%)

eGFR (mL/min/1.73 m^2^)	98 (35)

Chronic kidney disease	447 (8.2%)

UACR (mg/g)	6.7 (9.3)

Albuminuria	772 (14.2%)

Blood Cd (mcg/L)	0.40 (0.38)

Urinary Cd (mcg/g)	0.29 (0.35)

Urinary Cd levels were significantly higher in smokers (0.39 (0.46) mcg/g) compared with non smokers (0.23 (0.25) mcg/g) (*P *< 0.001). Blood Cd levels were significantly higher in smokers (0.60 (0.52) mcg/L) compared with non smokers (0.30 (0.20 mcg/g) (*P *< 0.001). Furthermore, there was a positive correlation between blood cotinine, a metabolite of nicotine, and both blood Cd (r = 0.44, *P *< 0.001) and urinary Cd (r = 0.12, *P *< 0.001).

Urinary Cd was higher in females (0.33 (0.38) mcg/g) compared with males (0.25 (0.32) mcg/g) (*P *< 0.001). Blood Cd levels were similar between females (0.40 (0.33) mcg/L) and males (0.40 (0.50) mcg/L) (*P *= 0.137).

Urinary and blood Cd were significantly related with UACR (r = 0.22 for urinary Cd, r = 0.09 for blood Cd) and eGFR (r = -0.32 for urinary Cd, r = - 0.15 for blood Cd) (*P *< 0.001 for each correlation).

Urinary Cd was higher in subjects with CKD (0.40 (0.38) mcg/g) compared with subjects without CKD (0.28 (0.35) mcg/g) (*P *< 0.001) and in subjects with ALB (0.38 (0.44) mcg/g) compared with subjects without ALB (0.27 (0.34) mcg/g) (*P *< 0.001).

Blood Cd was higher in subjects with CKD (0.50 (0.40) mcg/L) compared with subjects without CKD (0.40 (0.40) mcg/L) (*P *< 0.001) and in subjects with ALB (0.50 (0.40) mcg/L) compared with subjects without ALB (0.50 (0.40) mcg/L) (*P *< 0.001).

The logistic regression analysis of the association between blood and urinary Cd and CKD and ALB adjusted for age, gender, race/ethnicity and BMI showed a significant association between blood Cd levels > 1 mcg/L and both CKD (OR 1.48, 95% CI 1.01, 2.17) (*P *= 0.046) and ALB (OR 1.41, 95% CI 1.10, 1.82) (*P *= 0.007); a significant association was also found between urinary Cd levels > 1 mcg/g and ALB (OR 1.63, 95% CI 1.23, 2.16) (*P *= 0.001) but not CKD (OR 0.70, 95% CI 0.47, 1.04) (*P *= 0.079).

**Figure 1:**
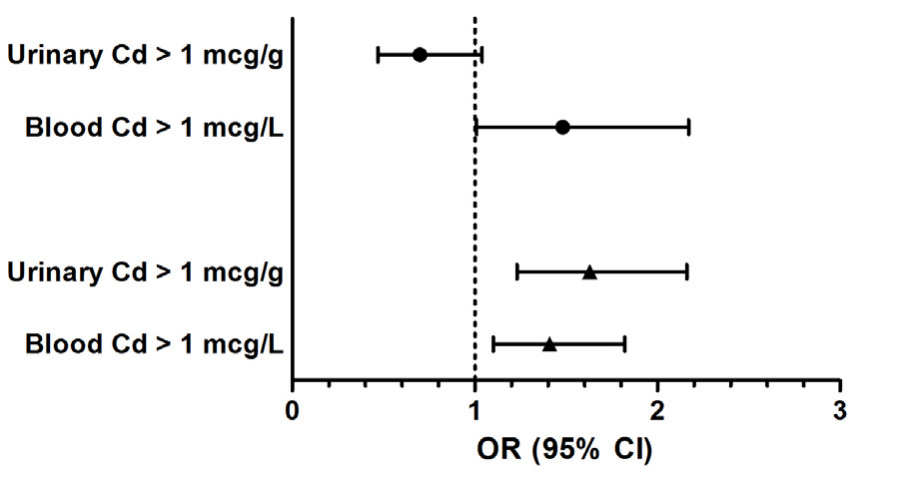
**Odds Ratios for CKD and ALB by levels of urinary and blood Cd**. Circles represent CKD, triangles represent ALB.

The results of the univariate and multivariate regression models, including an analysis of the interaction effect of urinary and blood Cd are shown in Table 2 and Figure 1.

**Table 2:** Results of the multivariate logistic regression

	CKD			ALB		
	**OR**	**95% CI**	**Sig. (*P*)**	**OR**	**95% CI**	**Sig. (*P*)**

**Urinary Cd - univariate model**						

Urinary Cd						
- ≤ 1 mcg/g	Ref	Ref	-	Ref	Ref	-
- > 1 mcg/g	1.61	1.14, 2.28	0.007	2.23	1.72, 2.89	< 0.001

**Urinary Cd - multivariate model**						

Urinary Cd						
- ≤ 1 mcg/g	Ref	Ref	-	Ref	Ref	-
- > 1 mcg/g	0.70	0.47, 1.04	0.079	1.63	1.23, 2.16	0.001

Age (1 year)	1.12	1.11, 1.13	< 0.001	1.04	1.03, 1.04	< 0.001

Gender						
- Female	Ref	Ref	-	Ref	Ref	-
- Male	1.28	1.01, 1.62	0.039	0.65	0.55, 0.77	< 0.001

BMI (1 Kg/m^2^)	1.03	1.01, 1.05	0.002	1.04	1.03, 1.05	< 0.001

Race/ethnicity						
- Mexican american	Ref	Ref	-	Ref	Ref	-
- Non-hispanic white	3.50	2.00, 6.11	< 0.001	1.16	0.84, 1.62	0.371
- Non Hispanic black	2.65	1.82, 3.86	< 0.001	0.65	0.53, 0.80	< 0.001
- Other	1.89	1.20, 2.97	< 0.001	1.12	0.89, 1.42	0.325

**Blood Cd - univariate model**						

Blood Cd						
- ≤ 1 mcg/L	Ref	Ref	-	Ref	Ref	-
- > 1 mcg/L	0.96	0.70, 1.32	0.007	1.28	1.02, 1.61	0.034

**Blood Cd - multivariate model**						

Blood Cd						
- ≤ 1 mcg/L	Ref	Ref	-	Ref	Ref	-
- > 1 mcg/L	1.48	1.01, 2.17	0.046	1.41	1.10, 1.82	0.007

Age (1 year)	1.12	1.11, 1.13	< 0.001	1.04	1.03, 1.04	< 0.001

Gender						
- Female	Ref	Ref	-	Ref	Ref	-
- Male	1.22	0.97, 1.54	0.094	0.67	0.56, 0.79	< 0.001

BMI (1 Kg/m^2^)	1.04	1.02, 1.06	< 0.001	1.04	1.02, 1.05	< 0.001

Race/ethnicity						
- Mexican american	Ref	Ref	-	Ref	Ref	-
- Non-hispanic white	3.29	1.88, 5.76	< 0.001	1.15	0.83, 1.60	0.405
- Non Hispanic black	2.66	1.83, 3.88	< 0.001	0.64	0.52, 0.79	< 0.001
- Other	1.88	1.19, 2.95	0.006	1.10	0.87, 1.38	0.456

**Interaction urinary/blood Cd**						

Urinary/blood Cd						
- ≤ 1 mcg/g-1 mcg/L	Ref	Ref	-	Ref	Ref	-
- > 1 mcg/g-1 mcg/L	1.01	0.60, 1. 27	0.964	1.62	1.10, 2.38	0.014

**Figure 2:**
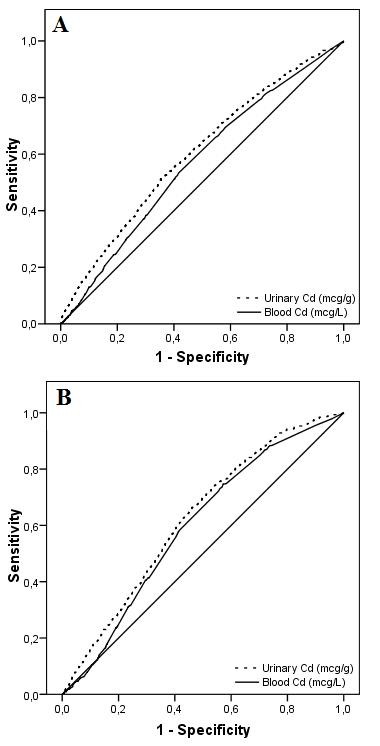
**ROC curves for ALB and CKD by levels of urinary and blood Cd**. ROC curves for ALB (A) and for CKD (B) by levels of urinary and blood Cd.

Finally, receiver operating characteristic (ROC) curves were performed to test the predictivity of urinary and blood Cd levels with regard to presence of CKD and ALB (Figure 2).

The areas under the ROC (AUROC) curve for CKD were 0.62 for urinary Cd (95% CI 0.60, 0.65) and 0.60 (95% CI 0.57, 0.62) for blood Cd. Urinary Cd showed a sensitivity > 70% for levels ≥ 0.27 mcg/g and a specificity > 70% for levels ≥ 0.44 mcg/g, while blood Cd showed a sensitivity > 70% for levels ≥ 0.39 mcg/L and a specificity > 70% for levels ≥ 0.55 mcg/L.

The areas under the ROC (AUROC) curve for ALB were 0.60 for urinary Cd (95% CI 0.58, 0.62) and 0.57 for blood Cd (95% CI 0.55, 0.59). Urinary Cd showed a sensitivity > 70% for levels ≥ 0.24 mcg/g and a specificity > 70% for levels ≥ 0.43 mcg/g, while blood Cd showed a sensitivity > 70% for levels ≥ 0.31 mcg/L and a specificity > 70% for levels ≥ 0.54 mcg/L.

## Discussion

Navas-Acien et al [8] have recently reported in the large 1999-2006 NHANES adult population, a representative sample of the general United States adult population, the existence of an association between renal dysfunction and blood Cd supporting previous findings that Cd is a nephrotoxin even at low level exposition [13]. In this study we confirm, in the subset of the same NHANES population who has urinary Cd data, that blood Cd is associated with impaired renal function and proteinuria.

Although blood Cd increases promptly following exposure, the blood Cd concentration is considered to be a less reliable marker of health effects than the urinary excretion [9], and actually most of the papers investigating renal toxicity by Cd have analyzed the urinary excretion of the metal [14]. It seems that the chronic accumulation of Cd in the renal cortex is responsible for the nephrotoxicity [15], and the cortical content is mirrored by the urinary excretion of Cd [16].

Present data shows the association of marginally high urinary Cd excretion with abnormal albuminuria, while no relationship was evidenced with CKD (eGFR < 60 mL/min/1.73 m^2^). ROC curve analysis shows a prediction of urinary Cd for both CKD and albuminuria.

Urine concentrations above 10 mcg/g of creatinine are considered evidence of excessive exposure to Cd [17], although the U.S. Occupational Safety and Health Administration considers 3 mcg/g of creatinine as the safety standard for urinary Cd. Very few Americans are exposed to such high levels of Cd: of the investigated subjects in the 1999-2006 NHANES surveys, no one had urinary Cd levels above 10 mcg/g and only 8 exceeded 3 mcg/g. However, our analysis suggest that even low urinary levels of Cd, an order of magnitude lower than those considered as toxic in industrial medicine, may have adverse effects on the kidney and a much higher number of Americans have such an urinary excretion of Cd (645 subjects [12.6%] had urinary Cd levels > 0.75 mcg/g in our study). The suspect that a mild exposition to Cd, as mirrored by urinary Cd levels in the same order of magnitude of the present one, is possibly responsible for renal dysfunction was raised in a small post-menopausal female population in Sweden [18]. Our study confirms that this may be the case, proposing Cd as a potential, relevant contributor to the epidemics of CKD observed nowadays.

An association between kidney disease and Cd was first noted at the end of the 19^th ^century, but the distinctive tubular proteinuria was not recognized until the 1940s [19]. For a long period, however, it remained unclear whether Cadmium nephropathy was the result of concomitant Lead nephropathy or of the effects of urinary tract obstruction. As a matter of fact, Cd workers are frequently exposed also to Pb that induces chronic tubulointerstitial nephritis, and kidney stones are common among Cd workers [20]. Nevertheless, the role of Cd as a nephrotoxin in humans is now widely acknowledged [21] and it is known that the relative mortality from kidney disease is increased among Cd workers [22]. Since in the 1999-2006 NHANES surveys no question regarded renal stones it was not possible to investigate whether nephrolithiasis played any role in the association between renal dysfunction and Cd levels.

A study on a sub-sample of the presently investigated population (NHANES 1999-2000, 2001-2002, 2003-2004) has been published which discloses the association of blood Cd, but not urinary Cd, with a modest elevation in blood pressure levels [7]. These data suggest that the relationship between blood Cd and CKD (as here defined, i.e. GFR < 60 mL/min/1.73 m^2^) is hemodynamic, thus highly susceptible to acute effects as those possibly depending on acute variations of blood Cd levels.

Besides industrial pollution, other sources of Cd exposure are cigarette smoke, ingestion of polluted vegetables, and ambient air in urban-industrialized areas [5,23,24]. The significant correlation we observed between blood levels of cotinine and Cd may suggest that cigarette smoking is responsible for such a low level exposure to Cd. Cigarette smoke contains about 4000 chemical substances, including toxic metals such as Cd. Smoking 20 cigarettes a day results in inhalation of an average 3.6-6.0 mcg of Cd [25]. It was shown that while the cigarette is burning, 30% of Cd and 11% of Pb present in the whole cigarette is released into the smoke; moreover, for Cd, as opposite to Pb, there is a high positive correlation between the metal content in cigarettes and tobacco and its release into the smoke; and finally, subjects smoking cigarettes containing the highest Cd amount have higher blood Cd concentration than smokers of low Cd content cigarette brands [26]. In Cd and Pb unpolluted areas, cigarette smoking may thus create a serious source of chronic exposure to these metals, especially to Cd [25].

The strong points of our study include the well-characterized NHANES datasets, large sample size, and U.S. nationally representative data. Since several potential confounders were collected as part of the NHANES, we were able to examine the association between Cd exposure and CKD and albuminuria - both well established risk factors for end stage renal disease in the general population [27] - after adjustment for potential confounders. Limitations include the fact that Cd urinary levels were low, close to the detection limit of the instrument.. Since the NHANES is a cross-sectional study it is not possible to infer causality between risk factors and CKD. Furthermore, the concept of chronicity of the renal dysfunction is vague since only a small subsample of the investigated NHANES population underwent a second evaluation of albuminuria. This may be relevant to the interpretation of present results. Actually we cannot confirm that the albuminuria found to be associated with urinary Cd levels is really chronic and irreversible. Typical markers of proximal tubular dysfunction such as β2-microglobulin, which is considered as one of the best indicators of Cd-induced nephropathy, could not be investigated since they were not collected during the NHANES. Since albumin is handled by the proximal tubule as a LMW protein, albuminuria per se could be a marker of a dysfunction in the proximal tubule machinery for LMW protein reabsorption [28] which may not constitute a "chronic kidney disease" condition. The Cd-metallothionein-1 complexes filtered through the glomerulus accumulate in the proximal tubule [4] where they interfere with the tubular function, and namely with the above machinery. In the typical Cadmium nephropathy, proximal tubular dysfunction persists until renal failure supervenes but this is considered to occur following much more relevant exposure to Cd. It is not known if low level exposure to Cd induces an irreversible dysfunction in tubular biology, more severe than the simple interference with LMW protein reabsorption, or reverts after giving up exposition to Cd. This interpretation requires experimental verification at these low levels of exposure to Cd.

In conclusion, data from the large general population of the NHANES suggest that the chronic exposition to low level Cd is associated with albuminuria, a well known marker of renal dysfunction and may concur in explaining the epidemics of chronic kidney disease.

## Conclusions

In a large sample of the general population, levels of urinary and blood Cd below the accepted thresholds are associated with a higher proportion of kidney disease and albuminuria.

## Competing interests

The authors declare that they have no competing interests.

## Authors' contributions

PMF participated in the design of the study, carried out the statistical analysis and participated in drafting the manuscript; SC participated in the design of the study; AN participated in drafting the manuscript; GG conceived the study, participated in its design and drafting and coordinated the research. All authors read and approved the final manuscript.

## Pre-publication history

The pre-publication history for this paper can be accessed here:

http://www.biomedcentral.com/1471-2458/10/304/prepub
